# Modelling Voluntary General Population Vaccination Strategies during COVID-19 Outbreak: Influence of Disease Prevalence

**DOI:** 10.3390/ijerph18126217

**Published:** 2021-06-08

**Authors:** Rastko Jovanović, Miloš Davidović, Ivan Lazović, Maja Jovanović, Milena Jovašević-Stojanović

**Affiliations:** “VINČA” Institute of Nuclear Sciences-National Institute of the Republic of Serbia, University of Belgrade, Mike Petrovica Alasa 12-14, P.O. Box 522, 11351 Vinca, Belgrade, Serbia; davidovic@vinca.rs (M.D.); lazovic@vinca.rs (I.L.); majaj@vin.bg.ac.rs (M.J.); mjovst@vin.bg.ac.rs (M.J.-S.)

**Keywords:** COVID-19, infectious diseases, modelling, graph theory, digital twinning, information spread, vaccination

## Abstract

A novel statistical model based on a two-layer, contact and information, graph is suggested in order to study the influence of disease prevalence on voluntary general population vaccination during the COVID-19 outbreak. Details about the structure and number of susceptible, infectious, and recovered/vaccinated individuals from the contact layer are simultaneously transferred to the information layer. The ever-growing wealth of information that is becoming available about the COVID virus was modelled at each individual level by a simplified proxy predictor of the amount of disease spread. Each informed individual, a node in a heterogeneous graph, makes a decision about vaccination “motivated” by their benefit. The obtained results showed that disease information type, global or local, has a significant impact on an individual vaccination decision. A number of different scenarios were investigated. The scenarios showed that in the case of the stronger impact of globally broadcasted disease information, individuals tend to vaccinate in larger numbers at the same time when the infection has already spread within the population. If individuals make vaccination decisions based on locally available information, the vaccination rate is uniformly spread during infection outbreak duration. Prioritising elderly population vaccination leads to an increased number of infected cases and a higher reduction in mortality. The developed model accuracy allows the precise targeting of vaccination order depending on the individuals’ number of social contacts. Precisely targeted vaccination, combined with pre-existing immunity, and public health measures can limit the infection to isolated hotspots inside the population, as well as significantly delay and lower the infection peak.

## 1. Introduction

The novel human coronavirus associated with severe acute respiratory syndrome (SARS-CoV-2) was first identified in December 2019, in Wuhan, China [1]. Infection caused by this new coronavirus (COVID-19) spread globally and the World Health Organization proclaimed it a pandemic on 11 March 2020 [2,3]. More than 106 million cases of COVID-19 including almost 2.5 million deaths were reported as of February 2021 [4].

The worldwide scientific community invested great efforts to find possible solutions to stop or, at least, mitigate the COVID-19 outbreak. Mathematical modelling and simulations have paramount significance in efforts to provide possible guidance and answers in a wide range of research fields [5].

B.S. Al-Anzi and co-authors fitted COVID-19 data for different countries to a regression model to derive future COVID-19 trajectories and their influence [6]. K. Rypdal and co-authors integrated equations of a standard Susceptible–Infectious–Recovered (SIR) model and performed country clustering to determine their response to changes in numbers of COVID-19 cases [7]. N. Anđelić and co-authors utilised an artificial intelligence-based genetic programming technique to derive a mathematical equation which can accurately predict the COVID-19 epidemic curve for the whole of the United States [8]. Y. Chen and co-authors used a multi-scale geographical weighted regression model to assess the urban resilience of 281 cities in China hit by the COVID-19 outbreak [9]. A.M.Y. Chu and co-authors suggested a novel latent pandemic space model for the analysis of global COVID-19 trends. The authors used the model to visualise the pandemic strain in time and space, and to assess the effectiveness of different measures for preventing infection spread [10]. R.O.J.H. Stutt and co-authors in their study [11] used two different models: a branching process transmission model and a modified (SEIR) model to determine the effectiveness of lockdown and mask-wearing policies for managing COVID-19 spread. A. Atangana suggested a modified SEIR model [12] based on Italian COVID data. Model capabilities were extended for a general application using novel fractal-fractional operators.

Several vaccines against SARS-CoV-2 are already available [13,14,15,16,17,18]. Mathematical modelling is already a proven tool for COVID-19 simulation, as discussed in the previous paragraphs. Thus, it is expected that numerical models can be very useful for forming strategies and guidelines for vaccine efficient distribution and application. However, as to the authors’ knowledge, there are only a few papers focused on vaccination against COVID-19.

B.H. Foy and co-authors in their paper [19] used a modified differential SEIR model with compartments corresponding to different age groups to investigate potential vaccination strategies. The authors concluded that the global recommendation to prioritise the elder population leads to the lowest number of deaths. It was also found that an increase in vaccine coverage decreases relative differences between different vaccination strategies. It should be noted that the work was limited to symptomatic and asymptomatic individuals belonging to different age groups. Different types of social contacts (at a workplace or inside households, etc.) were not in the scope of this work. Moreover, variable vaccine coverage and pre-existing immunity were not analysed.

K.M. Bubar and co-authors in their study [20] also adopted an age-based compartment SEIR model to investigate different vaccination strategies. The authors analysed the impact of different vaccine supply, vaccine rollout per-day, age-dependent vaccine efficiency, and pre-existing immunity on the cumulative number of infected, number of deceased, and years of life lost. It was concluded that highly efficient vaccines given to adults between 20 and 49 years of age minimise the cumulative number of infections. However, mortality is minimised when adults older than 60 years are prioritised. The use of serological tests to prioritise seronegative individuals’ vaccination led to lower COVID-19 transmission rates. It is important to underline that all input parameters (vaccine efficiency, serostatus) were constant inside one age group.

L. Matrajt and co-authors in their work [21] suggested a deterministic SEIR model with compartments corresponding to different age groups to identify the best possible strategy for general population vaccination. The model showed that when using lower efficiency vaccines, the number of deceased individuals is minimised if the elderly population (age over 65 years) is prioritised. However, in the case of high-efficiency vaccines, the suggested strategy is to prioritise the younger adult population to minimise the number of infected individuals. Detailed social contact structure investigation and its influence on vaccination strategy were out of the scope of this work.

M. Kohli and co-authors presented the results of their work [22] in which they used a Markov cohort model to determine vaccination cost. The population was divided into subgroups based on individuals’ age, age and risk, and age and occupation. Outcomes of different vaccination supplies were simulated over one year. The calculated results showed that the incremental cost-effectiveness ratio is higher than USD 50,000 per quality-adjusted life-year.

Previously described vaccination simulations are based on the SEIR deterministic differential models. These models offer valuable insights into possible vaccination strategies and can investigate a large number of different scenarios in a small amount of computation time. However, their main limitation is that all individuals in a single compartment have the same, constant characteristics.

The main aim of this work is to present a novel model for vaccination strategy simulation. While the model was developed with COVID-19 vaccination as an immediate use case, it could be useful in a number of additional scenarios. The non-exhaustive list includes later revaccination strategies, studies of information propagation of other public health-relevant information products such as air pollution data and their influence on daily habits, and similar. These kinds of models may be further refined to be incorporated into digital twin models, but can also bring valuable insight without incorporating them into full-fledged digital twin models. Digital twinning is a novel paradigm applicable in many sectors, including healthcare.

The proposed model uses a two-layer graph (contact and information) to describe a detailed population structure depending on the individuals’ age, household size, daily social behaviour, and varying vaccination (global and local) opinion. The detailed graph structure enables in-depth insight into possible vaccination scenarios, not possible using simpler compartment models.

The main subject of this work is the simulation of disease prevalence on general population vaccination during the COVID-19 outbreak.

## 2. Materials and Methods

### 2.1. Two-Layer Graph—Contact Layer

**Figure 1:**
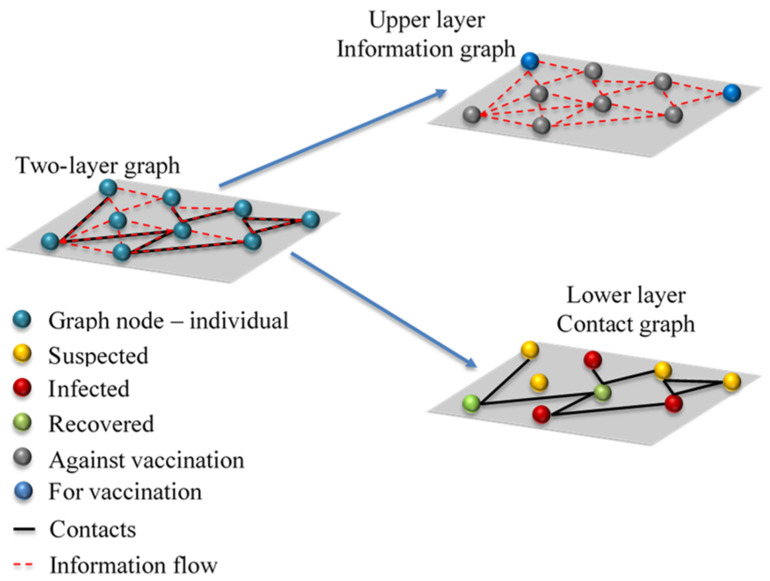
Schematic representation of two-layer graph composed of information and contact graphs. Left side—interplay between contact and information layers, top right corner—information layer, red line represents information flow, grey circles represent individuals against vaccination, and blue circles represent individuals opting for vaccination, bottom right corner—contact layer, black lines represent infection vectors, yellow circles represent suspected individuals, green circles represent recovered and/or vaccinated individuals, and red circles represent individuals in one of the infection stages.

The mathematical model is based on the two-layer graph. The lower layer is the contact graph, which characterises all personal contacts between different individuals in the considered community (Figure 1). The main assumption is that the epidemic outbreak time scale is much shorter than the time scale of events (births, natural deaths, long-term movements) which could impact contact graph structure.

The graphs based on real-life contacts are quite complex and require significant time for construction. However, they also offer a higher degree of realism compared with simpler graphs (random graphs, scale-free graphs) [23], and mathematical analysis on these graphs does not require extensive simulation [24,25]. Thus, a contact graph was adopted in this work.

**Figure 2:**
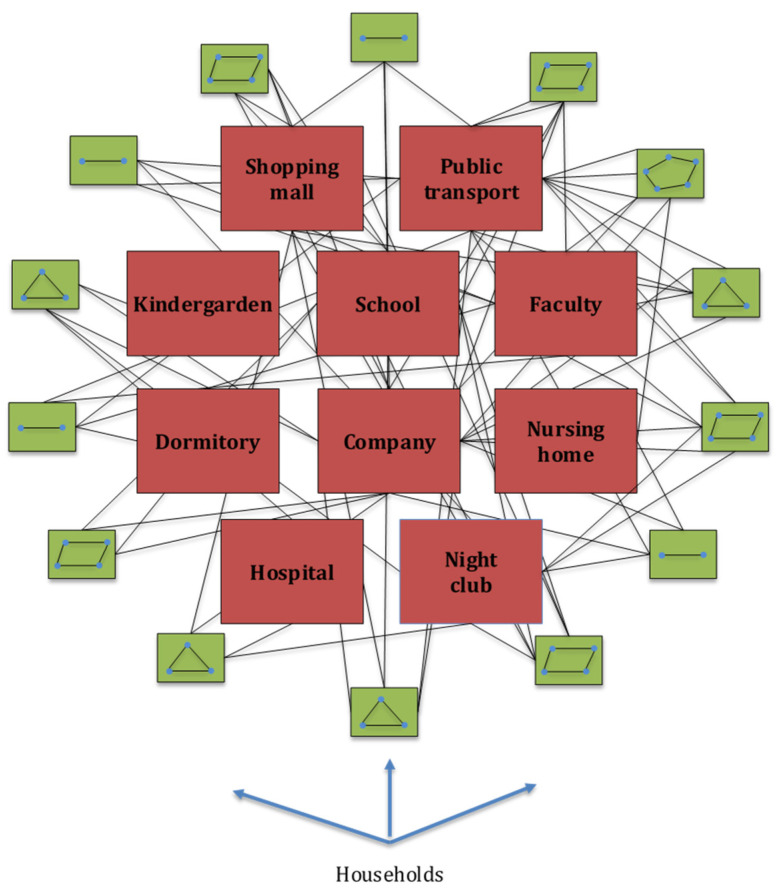
Simplified representation of contact graph layer: blue circles represent graph nodes (modelled individuals), green squares represent different structure and size households, red squares represent places for possible social contacts, and black lines represent graph edges—possible contact routes, i.e., possible infection vectors.

Each individual in the contact graph is represented by a single node. Contacts between different individuals are represented by graph edges. The initial graph structure is generated based on social data for the city of Belgrade, Republic of Serbia (RS). Belgrade is the capital city of RS with a population of 1,687,130 inhabitants in the metropolitan area [26]. One thousand households were sampled from the Belgrade household size distribution, leading to a total of 2730 graph nodes [27]. More specifically, the structure of households, number of household members, ratio of the number of households to the number and size of schools, companies, transport capacities, restaurants and clubs and other public facilities were determined in accordance with the latest census data for the city of Belgrade and Republic of Serbia [26,27,28,29,30,31,32,33]. A comparable number of graph nodes was used for simulating the SARS outbreak in the city of Vancouver, Canada, which has a similar population size [34]. Graph nodes are grouped in single-, two-, three-, four-, five-, six-, and six and more-member households according to [27]. Ages are assigned to all household members (graph nodes) according to age distribution obtained from [28]. Each household member is then assigned, according to their age and social status, to one of the following locations: kindergartens, primary schools, secondary schools, faculties, student dormitories, nursing homes for the elderly population, companies, hospitals, public transport, restaurants (pubs, night clubs), shopping malls, and other public places, as can be seen in Figure 2. The number and structure of these locations were adopted from publicly available datasets for the city of Belgrade [29,30] and RS [31,32,33]. A simplified representation of the contact graph is presented in Figure 2.

The model assumes that the infection spreads through weighted graph edges assigned randomly to each location. Edge weights represent probabilities that susceptible individuals will become infected due to contacts with infectious individuals.

Note that the direct route of infection between individual households was not included in the model, since during the whole duration of the modelled 3rd wave of COVID-19 infection, restriction of individual gatherings between members of different households was enforced. While there are no edges in the contact graph that directly model individual household interactions in the context of household gatherings, the spread vector between households still effectively exists via interaction of individuals at schools, public transport, shopping malls, workplaces, etc. The following edge weights were used in this work: 1 for households; 0.3 for kindergartens, primary schools, secondary schools, faculties, hospitals, and restaurants (pubs, night clubs); 0.65 for student dormitories and nursing homes; 0.03 for working places; and 0.003 for other public locations. Edge weights were adopted based on work [34]. The values for graph edge weights were additionally analysed using a trial and error procedure and showed a high degree of robustness to small changes in their values.

### 2.2. Two-Layer Graph—Information Layer

The upper layer of the two-layer graph is the information layer. Each individual receives either local or global information about COVID-19 infection propagation, modelled on the contact graph. Local information is represented by the number of infected nearest neighbours of the particular individual and global information is represented as the number of total infected cases in the whole simulated network of contacts. In real life, these may be interpreted as person to person communication in the case of local information, and mass media communication (TV and/or internet networks) in the case of global information. The individuals are also informed about the amount of protection in their neighbourhood. This effect is modelled as the number of immune nearest neighbours, either recovered or vaccinated. Thus, each individual (represented as a graph node) can make a situation-aware voluntary decision about his/her vaccination. In real life, people weigh costs and benefits in making a strategic decision about their vaccination. Costs are usually related to real or perceived vaccine side effects and efforts in the vaccination process.

The temptation to obtain protection from herd immunity generated by vaccinated neighbours also plays a significant role in vaccination cost. However, if a significantly large number of people employ this strategy, it will lead to low vaccination uptake and, consequently, prolong the existence or delay the start of a new COVID-19 outbreak. Thus, individuals’ decision-making becomes a vaccination game in which each person tries to maximise their own goal. It is important to underline that, unlike in compartment epidemic models, the heterogenic structure of the suggested model, based on graph theory, leads to different outcomes for each individual. Moreover, the outcome for the individual under consideration also changes in time, with epidemic spread and status (infected, recovered, vaccinated) of its neighbours.

**Figure 3:**
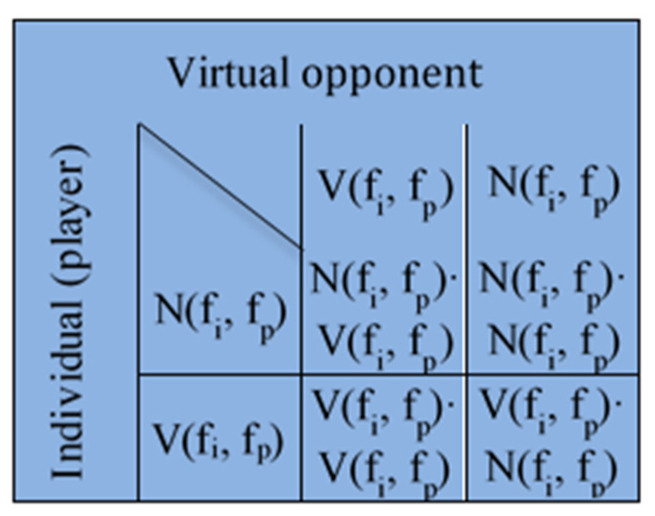
The payoff matrix in the vaccination game. *V* designates vaccination payoff, *N* designates anti-vaccination payoff.

The decision process is modelled using game theory. Each individual, modelled as a graph node, plays the game with a virtual opponent who has identical information and decision-making characteristics (Figure 3).

*V* is vaccination payoff and *N* is anti-vaccination payoff defined as functions of *f_p_* and *f_i_*. *f_p_*(*j*) is the fraction of the first neighbours of an individual represented by the node *j* that is immune, and *f_i_*(*j*) is defined by the following equation:

(1) $$f_{i}\left(j\right)=\alpha \frac{I}{N_{tot}}+\left(1-\alpha \right)\frac{n_{i}\left(j\right)}{n\left(j\right)}$$

where α∈[0,1] is a social parameter, which describes the influence of local and global information about disease prevalence in vaccination decision-making, *I* is the total number of infected individuals in the simulated population, *N_tot_* is the total size of the simulated population, *n_i_*(*j*) is the number of infected nearest neighbours of individual *j*, and *n*(*j*) is the total number of nearest neighbours of individual *j*. Small values of *α* indicate the prevailing influence of local information in vaccination decision-making, and the opposite, bigger values of *α* point out the stronger influence of global information.

Functions *NV*, *VN*, *NN*, *VV* are given by the following expressions:

(2) $$\begin{array}{l} NV=a\cdot f_{p}+b\;NN=c\cdot f_{p}+d\\ VN=e\cdot f_{i}+f\;VV=g\cdot f_{i}+h \end{array}$$

Equation (2) shape and constants *a*, *b*, *c*, *d*, *e*, *f*, *g*, and *h* are taken from the work [35].

The expression for the probability that the individual *j* will choose the vaccination scenario is given by the equation:

(3) $$p_{vacc}\left(j\right)=\frac{NN-VN}{\left(VV-NV\right)+\left(NN-VN\right)}$$

It is important to note that a piece of negative information can become prevailing if, for example, the particular local individual (agent) is surrounded by a high fraction of already immune, recovered or vaccinated individuals, thus making the individual susceptible to vaccine hesitancy and contributing to low vaccination probability.

### 2.3. Epidemic Model on Contact Graph

A modified SEIR-V model was developed and applied for COVID-19 infection spread in the framework of this study. The standard SEIR model assumes that each individual inside the observed population belongs to one of the following groups: susceptible (still has not had the disease, can become infected, and cannot spread disease), exposed (infected, but not yet infectious, and cannot spread disease), infectious (infected and can spread disease), recovered/removed (cannot become infected and cannot spread disease). The proposed model, based on [36], merges exposed and infected groups into a single category characterised by the disease’s infectiousness. Each individual from this new group is assigned state, *I*_1_···*I_Ntot_*, corresponding to their current daily stage of the disease infectiousness. COVID-19 infectiousness daily values were adopted from experimental data published in [37].

The likelihood that individual *j* will be infected due to contact with its first neighbours, in the probability theory [38,39], is expressed as:

(4) $$p_{infect}\left(j\right)=\sum_{i}\omega_{ij}\kappa_{stage,i}$$

where *p_infect_*(*j*) is the probability that individual *j* will become infected, *ω_ij_* is the graph adjacency matrix with dimensions *N_tot_*·*N_tot_*, and *κ_stage,i_* is the vector of infectiousness state *I*. The adjacency matrix, *ω_ij_*, is composed of averaged edge weights for each graph node.

All nodes are set to be susceptible at the beginning of the simulation and one node is set to be infected to simulate epidemic start. The first infected node is chosen randomly from the group of employed adults. This group was chosen based on the fact that its members have a large number of contacts and thus it is likely that only one infected individual would cause an epidemic outbreak. Moreover, the first discovered patient infected with COVID-19 in RS was an employed adult male [40].

The workflow of the COVID-19 model can be summarised as follows (Supplementary Material File S1, Figure S1). The outer Monte-Carlo loop is started with graph initialisation with all susceptible and one infectious node and all edges. The inner, SEIR-V loop is started next. A vector holding total and cumulative numbers of susceptible, infectious, and recovered and a vector holding states for all graph nodes are pre-allocated at the beginning of the inner loop. Equation (3) is calculated for all graph nodes. If the probability for vaccination is higher than the randomly generated number between zero and one, the node is assumed vaccinated. Equation (4) is calculated next for all susceptible nodes. The first stage of infectiousness is assigned to the nodes with the probability of infection higher than the random number in the range between zero and one. The state for all infectious nodes is advanced for one step. Nodes that are at the end of the infectiousness period are marked as recovered. A probability of 0.9 is assigned to recovered nodes. If this probability is higher than the random number between zero and one, recovered nodes will remain recovered. In the opposite case, the recovered node again becomes susceptible for infection. This correction factor incorporates the effect of infection and reinfection in the vaccinated population. The vectors which hold individual states and numbers of susceptible, infectious, and recovered are updated. Graph edges assigned to public places (public transport, nightclubs, shopping malls…) are updated to simulate the dynamic behaviour of the system. The inner loop is advanced until only vaccinated and recovered nodes remain. The outer loop is advanced until the defined number of Monte-Carlo steps equal to 5 × 10^5^ is reached. Results obtained for all Monte-Carlo iterations are averaged at the end of the SEIR-V simulation.

## 3. Results

### 3.1. Model Validation

**Figure 4:**
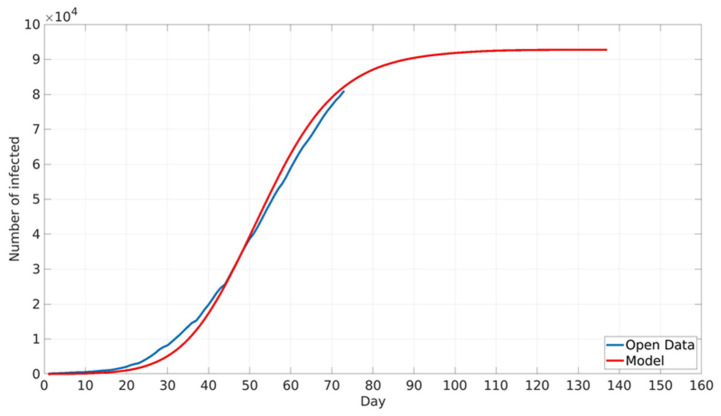
Comparison between cumulative numbers of reported real-life and modelled COVID-19 cases. Real-life data are marked with the blue line, modelled data are marked with the red line.

The suggested model was validated by comparison with the daily number of registered COVID-19 cases in Belgrade, RS. Daily values of COVID-19 cases in the period from 13 October 2020 (which corresponds to the start of the third wave of disease outbreak) to 3 December 2020 were obtained from open datasets provided by the National Public Health Institute of RS [41]. The model was not compared with COVID-19 data after the beginning of December because this period corresponds to the return of Belgrade citizens from Serbian ski centres. The Serbian crisis response team identified this as one of the main drivers for a new increase in COVID-19 infection intensity [40]. However, these types of imported contacts are beyond the scope of the presented work since the proposed model does not take into account long-term movements to and from Belgrade, as already stated. Furthermore, since one of the main points of the paper was various vaccination scenarios, and there were no viable vaccines at the time of the first two waves, such scenarios were not considered. However, in principle, the proposed model could be applied to any wave provided the accurate initial conditions for the model (sufficient number of tested individuals allowing for correct seeding of the model). A comparison between cumulative numbers of real-life and modelled COVID-19 cases is shown in Figure 4. It can be seen that the model closely predicts real-life epidemic spread, with a maximum relative error of about 7%. The obtained model accuracy is satisfactory, taking into account the model’s assumptions [42].

### 3.2. Influence of Different Social Factor Values

**Figure 5:**
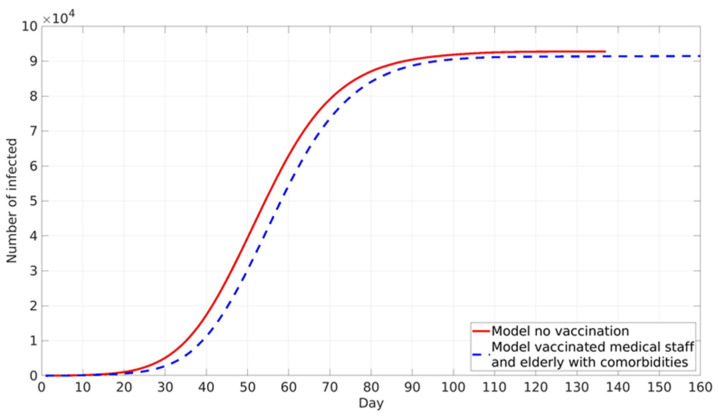
The model predicted cumulative numbers of infected cases: without vaccination—red line, vaccinated healthcare workers in COVID system and elderly population in nursing homes—blue dotted line.

Vaccination against COVID-19 in RS is voluntary. However, disease prevalence did not play a major role in the vaccination of the two following groups. Health workers in COVID zones are directly in contact with COVID-19 patients, and thus their vaccination hesitancy is very low due to high occupational risk. The second group is elderly individuals in nursing homes, who, due to their age and pre-existing comorbidities for COVID-19, have higher risks of serious illness upon infection and thus opted for vaccination in large numbers. Accordingly, it was assumed that these two groups were vaccinated first at a constant rate independent of local and global information spread (Figure 5). 

Vaccination of these two high-risk groups does not have a high impact on epidemic spread, as is expected due to the low absolute number of vaccinated individuals. The final cumulative number is lower by about 1% compared with the case without vaccination. A somewhat stronger influence is present in epidemic peak delay, which is shifted for 5 days in the vaccination scenario (Figure 5).

**Figure 6:**
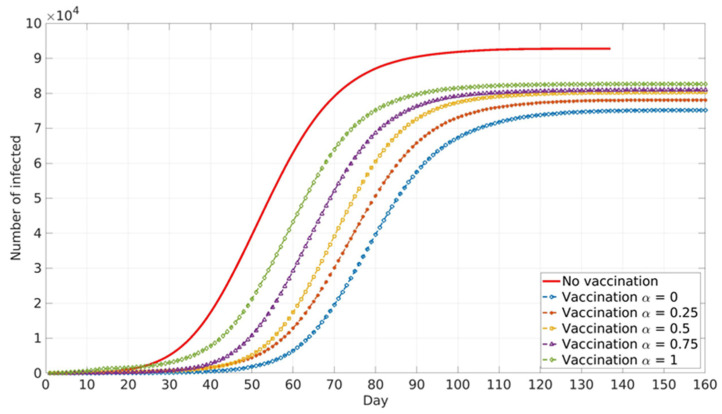
Vaccinated healthcare workers in the COVID system and adults over 65 years. The model predicted cumulative numbers of infected cases depending on social parameter α.

**Figure 7:**
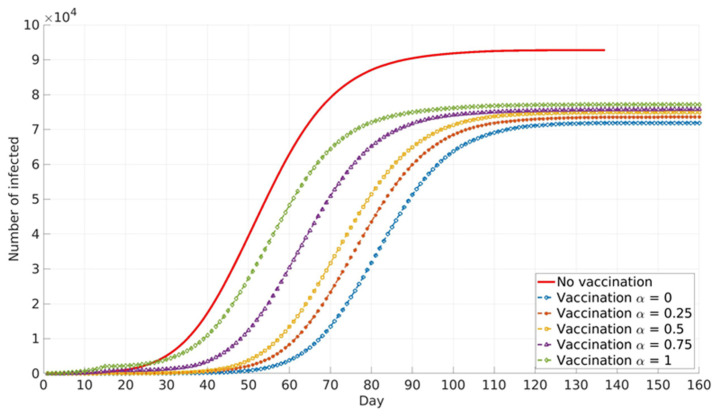
Vaccinated all healthcare workers, adults over 65 years, and adults from priority groups. The model predicted cumulative numbers of infected cases depending on social parameter α.

**Figure 8:**
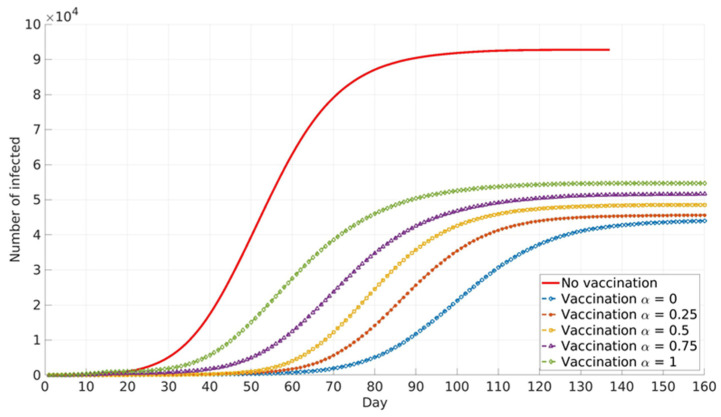
Vaccinated general adult population. The model predicted cumulative numbers of infected cases depending on social parameter α.

The impact of the social parameter, α, which describes the influence of local and global information in vaccination decision-making, on the cumulative number of infected cases for different population groups is presented in Figure 6, Figure 7 and Figure 8.

Similar trends are presented for all age groups (Figure 6, Figure 7 and Figure 8), and the number of cumulative cases increases with the increase in α. This can be explained by the fact that global information spread dominates at higher α values. In this case, individuals’ awareness of disease dangers and vaccination benefits is low until a large portion of the total population is affected by COVID-19 infection. Consequently, individuals tend to opt for vaccination when infection spread inside the population is already advanced. In this case, large numbers of individuals decide to vaccinate at the same time (near the peak of infection). If this number overweighs vaccination capacity, vaccination will be delayed and will not reach its full potential. Local information plays a dominant role at lower α values and this seems to benefit vaccination and lowers the number of infected individuals inside the population. This may be explained by the fact that individuals’ awareness rises almost at the same time with the local increase in infection in their immediate neighbourhood. Thus, vaccination coverage is more uniformly distributed across the population, both in time and in space.

Relative differences in the cumulative number of infected cases corresponding to different α values increase with the increase in vaccination coverage. This may contribute to the fact that all the above-described effects intensify with the rise in the number of individuals faced with vaccination dilemma.

### 3.3. Variable Social Factor Simulations

In real life, people make different decisions based on their social position, age, economic status, etc. This effect was incorporated into the suggested model, implementing different α values for different age groups during general population vaccination. The social factor, α, values were assigned based on a recent public survey which showed that about 40% of Serbian citizens do not trust media information and only about 20% have limited or complete trust in public media. The survey showed that younger adults between 20 and 40 years of age have the lowest or no trust in public media information [43]. Based on the survey, the following α values were adopted in this work: 0.75, 0.5, 0.25, and 0 for retired, unemployed, employed, and student population, respectively.

Two different scenarios, with variable α values, were calculated in the next phase of simulations. The first scenario, (1a), aims to minimise the number of deceased individuals, prioritising the vaccination of the elder population (years of age > 65), who are more likely to develop severe COVID-19 infection. The second scenario, (2a), aims to minimise the number of infected individuals, prioritising the vaccination of groups with a large number of contacts (working-age population and student population). The obtained results are shown in Supplementary Material File S2 (Figures S2 and S3).

The high accuracy of the constructed two-layer graph allowed for further simulation refinement. To obtain more in-depth insight into general population vaccination, two additional scenarios were simulated. Again, the first scenario, (1b), aims to minimise the number of deaths, and the second scenario, (2b), aims to minimise the number of infected individuals. The main difference is that the younger adult population was vaccinated randomly in the previous scenarios ((1a) and (2a)). In the new scenarios ((1b) and (2b)), the vaccination of the younger adult population is performed in the order of the number of their social contacts at workplaces or faculties (dormitories). The obtained results for scenarios (1b) and (2b) are shown in Supplementary Material File S2 (Figures S2 and S3).

### 3.4. The Variable Social Factor, Simulation of Pre-Existing Immunity Influence

A recent study about pre-existing immunity against COVID-19 was performed in RS by the Institute for Epidemiology and the Institute for Application of Nuclear Energy, [44,45]. The study reported that between 20% and 30% of the population in RS have antibodies against COVID-19. However, due to the low population response for participation in the study, this conclusion has to be considered with great care. The influence of pre-existing immunity on general population vaccination against COVID-19 was conducted in the next phase of this work. Simulations were performed assuming pre-existing immunity in the 0% scenario (3a), 10% scenario (3b), 20% scenario (3c), and 30% scenario (3d) of the total population in Belgrade, RS. 

**Figure 9:**
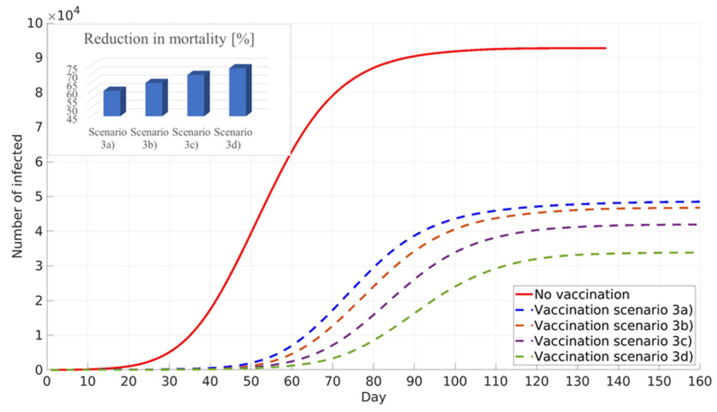
Influence of pre-existing immunity on the cumulative number of infected and reduction in mortality. General population vaccination scenario—minimised number of deceased.

All simulations in scenario three were performed to minimise COVID-19 deaths, prioritising elder population vaccination (Figure 9). An increase in the percentage of the population with pre-existing immunity leads to a decrease in infection numbers, delay of the infection peak, and decrease in COVID-19 deaths.

**Figure 10:**
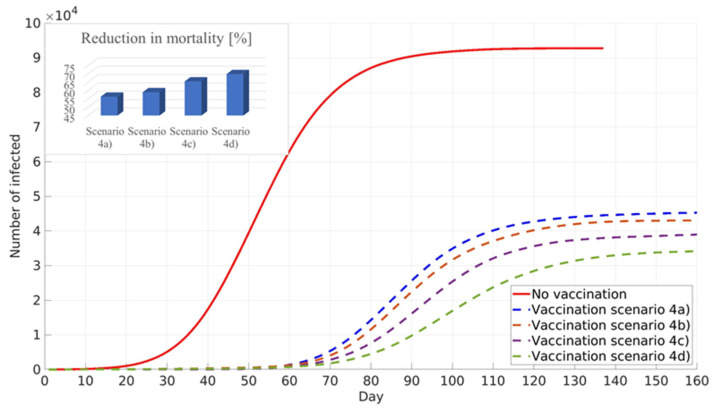
Influence of pre-existing immunity on the cumulative number of infected and reduction in mortality. General population vaccination—minimised number of infected.

All simulations performed for scenario four target the minimisation of numbers of infected individuals, assuming pre-existing immunity: 0% scenario (4a), 10% scenario (4b), 20% scenario (4c), and 30% scenario (4d) of the total population in Belgrade, RS. The simulation results are shown in Figure 10. It can be seen that similar to the previous scenario, an increase in the pre-existing immunity percentage leads to a decrease in the total number of infected cases, delay of the infection peak, and reduction in COVID-19 mortality. It should be noted that, due to different vaccination prioritisation strategies, cumulative numbers of infected cases and the reduction in COVID-19 deaths are lower in scenario four than in scenario three (Figure 9 and Figure 10).

### 3.5. The Variable Social Factor, Simulation of Pre-Existing Immunity and Prioritised Vaccination of Younger Population

**Figure 11:**
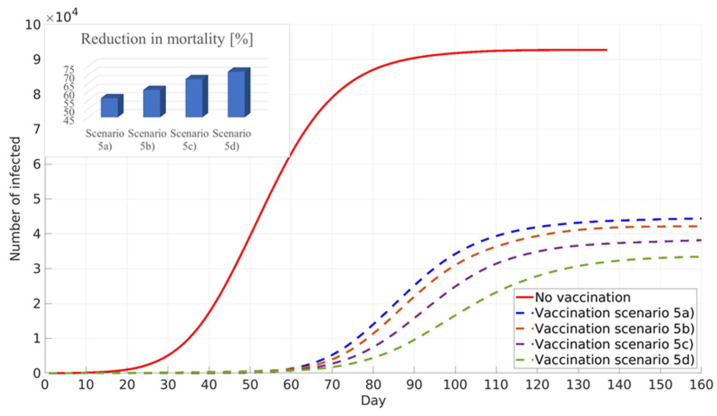
Targeted vaccination of younger population—minimised number of infected.

This new scenario prioritises the vaccination of the younger population, and the obtained results showed similar numbers of infected individuals as in the scenario in which the vaccination strategy prioritised adults with a large number of contacts. However, the new scenario showed an increase in mortality reduction, which can be explained by the fact that vaccinated young adults are not able to transfer disease to the elder population in their households, Figure 11.

### 3.6. The Variable Social Factor, Simulation of Pre-Existing Immunity and Lockdown Measures Influence

**Figure 12:**
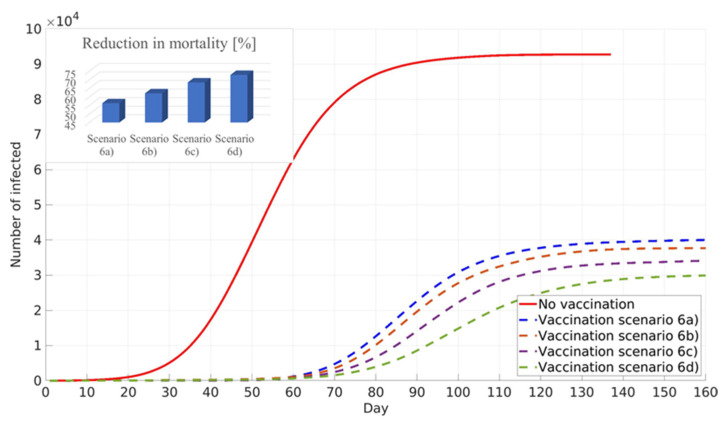
Influence of the imposed lockdown measures on the cumulative number of infected and reduction in mortality. General population vaccination—minimised number of infected.

The next calculated scenario investigates the influence of lockdown policies on the vaccination of the population with pre-existing immunity. Values of the social factor and the fraction of the population with developed immunity are the same as in the previous scenarios. It can be seen that lockdown measures lead to a further decrease in infected individuals and a further reduction in mortality (Figure 12).

The timescale present in the graphs refers to the timescale of the modelled 3rd wave of COVID-19 in the city of Belgrade. At the beginning of the 3rd wave (unlike the beginning of the 1st and 2nd wave), vaccines were already in the final stages of validation. Thus, from the standpoint of vaccination strategy development, it is relevant to evaluate the implications of the models of vaccination immediately following COVID-19 outbreaks from limiting the initial number of cases.

**Figure 13:**
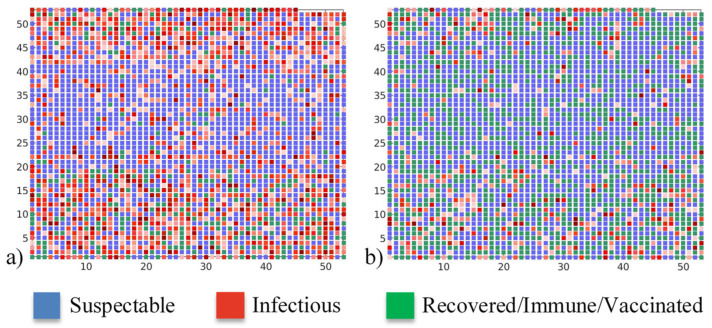
Daily numbers of susceptible, infectious, and recovered/immune/vaccinated at the peak of the epidemic. Case: (**a**) no vaccination, (**b**) general population contact-based vaccination with 30% of pre-existing immunity. Axis numbers represent graph nodes (modelled individuals), each square corresponds to a single graph node.

Clearer insight into epidemic spread can be obtained from the daily number of susceptible, infectious, and recovered/immune/vaccinated individuals at the peak of the epidemic, presented in Figure 13. It can be seen that pre-existing immunity with a proper vaccination strategy can both delay and lower the intensity of the COVID-19 peak. If there is no pre-existing immunity and vaccination, the epidemic reaches its maximum in 52 days, affecting almost half of the population and forming a spanning cluster. However, when there is significant pre-existing immunity in the population together with an efficient vaccination campaign, the epidemic intensity is significantly lowered, and less than 3% of the population is affected. Moreover, the epidemic is not spread over the whole graph and, instead, isolated local pockets of infected individuals are observed. In the case of 30% of the population with pre-existing immunity, and the vaccination strategy of minimising infection numbers, the epidemic reaches its peak after 93 days.

## 4. Discussion

COVID-19 had a devastating impact both on human health and the economy. Vaccines against novel SARS-CoV-2 were developed at an unprecedented rate and, thanks to their high efficiency, offer the best possible solution to stop the pandemic. The vaccination of high-priority groups (healthcare workers employed in COVID sectors, elderly individuals in nursing homes…) is already underway in many countries. However, parallel with this process, it is necessary to plan and define the best possible strategy for general population vaccination.

The major factor influencing any possible voluntary vaccination scenario is disease prevalence in the considered population. The novel framework based on two-layer graphs was suggested, validated, and used for the simulation of possible vaccination scenarios in the framework of this study. COVID-19 infection outbreak was modelled on the contact graph layer and information spread in the population was modelled on the information graph layer. The novel SEIR-V (Susceptible, Infectious, Recovered—Vaccinated) epidemic model was solved on a highly detailed contact graph. Information about the COVID-19 infection spread was transferred to each modelled individual. The informed individuals were faced with a cost–benefit vaccination choice dilemma modelled using game theory, in which each individual plays against a virtual opponent with the same characteristics.

Although more reliable results might be expected from deterministic models or stochastic models on finer graphs, the main aim of this work is to present a new modelling approach for the influence of global and local information spread on vaccination against COVID-19. The stochastic model, presented here, describes social contact structure and then introduces the information layer based on local and global COVID-19 infection numbers. The proposed modelling framework offers possibilities for further refinement, e.g., by introducing more detailed information about anti-epidemic health policies.

The impact of social factor, α, which determines the contribution of both local and global information in vaccination decision-making, was assessed in the next phase. It was found that the social parameter has a significant influence on the cumulative number of infected cases. This influence becomes stronger with the increase in vaccination uptake. Higher values of the social parameter correspond to the dominance of global information, and smaller values correspond to the dominance of local information in vaccination decision-making. The cumulative number of infected individuals has a sharper increase and higher final value when individuals’ decision-making is dominated by global information. This may be explained by individual real-life behaviour, in which they will not take vaccination action unless a significant portion of the total population is not infected. When global information about the high amount of infected cases is spread through the information graph, individuals tend to vaccinate in large numbers at once, which may cause the overstretching of the vaccination system. In the case when local information, obtained from the nearest neighbours, governs the vaccination decision, individuals tend to vaccinate as soon as the infection spreads in their proximity, with more uniform vaccination rates compared with the previous case.

The variable social parameter, based on population age, was incorporated into the model in the next phase of this work. Two different scenarios were investigated, one aiming to minimise the number of COVID-19 deaths by prioritising the vaccination of elderly individuals, and the second which tries to minimise the number of infected cases, prioritising the vaccination of younger adults, who likely have more social contacts. The obtained results showed that minimising the number of COVID-19 deceased leads to a higher number of infected individuals and vice versa. This outcome faces public health officials and other government bodies with a major decision about vaccination strategy adoption. It should be noted that incorporating more social groups, such as religious, gender, antivaxxer, and chronically sick, and increasing graph nodes and their connections would enable even more reliable results, but subject to the available computational resources.

Although the generated contact graph closely mimics the statistical description of important features of the city of Belgrade, a further increase in the graph size and refinement of the level of detail of the modelled phenomena would allow the incorporation of additional disease spread scenarios. In addition to the size of the contact graph, the model itself does not include imported cases, i.e., it assumes no inter-municipality travels, which does not fully replicate real epidemic behaviour. Further simplification of the model in the manuscript assumes no changes (dynamic behaviour) in public health policies for the considered time scale. During the time scale of the model, variables that change are numbers of susceptible, exposed, infected, and recovered individuals, disease prevalence and vaccination cost/benefit functions. Moreover, the fineness of the model does not allow the direct inclusion of very small groups of individuals with high vaccination risks, such as pregnant women, chemotherapy patients, etc. In the proposed model, such individuals are all aggregately included into random individuals for whom vaccination was ineffective.

High enough accuracy of the generated contact graph allowed for precise targeting of population groups which should be prioritised for vaccination. Younger adults (employed and students) were vaccinated in order of the number of their social contacts. This led to a further decrease in the cumulative number of infected individuals and a decrease in mortality caused by COVID-19, compared with previously investigated scenarios. However, the results again pointed out that minimising the number of deceased leads to an increase in the number of infected individuals and the other way round.

The influence of pre-existing immunity was investigated in the next phase of this work. It was shown that an increase in existing herd immunity lowers the numbers of infected and deceased during the COVID-19 outbreak. A combination of pre-existing immunity of 10–20% of the population under consideration with priority-based vaccination will not eliminate COVID-19 but will reduce global outbreak to local hotspots affecting a minor amount of the population.

In the last part of this work, an additional scenario which helps to elucidate the difference between mild and more strict public health measures was considered. The calculated scenario showed the significant influence of strict public health measures on the decrease in the number of infected individuals and on a further reduction in mortality.

## 5. Conclusions

The novel two-layer graph-based SEIR-V (Susceptible, Infectious, Recovered—Vaccinated) model was applied to determine the influence of disease prevalence during voluntary general population vaccination against SARS-CoV-2. The high reality of the generated information and contact graph layers enabled in-depth analysis of different vaccination scenarios.

It was found that if global information has a prevailing role during the COVID-19 outbreak, the population tends to decide to vaccinate instantly and in large numbers when the infection has already spread, which may lead to the overstretching of both vaccination and hospital systems. If the vaccination decision is based on local information, individuals decide to vaccinate based on the number of their infected nearest neighbours, which leads to more uniform and earlier vaccination.

Prioritising elderly population vaccination leads to a lower number of COVID-19 deaths and sharper increase in and higher final values of COVID-19 infection. Prioritising the vaccination of employed adults and the student population has the opposite effect, minimising the number of infected cases, delaying the infection peak, and lowering the decrease in deceased cases.

Precise targeting of individuals’ order of vaccination based on the number of their social contacts combined with pre-existing immunity in a population may significantly reduce COVID-19 infection spread, significantly delaying and lowering the infection peak. In this case, the disease outbreak is reduced to a small number of local hotspots.

Strict public health measures visibly contribute to lowering COVID-19 infection cases and a reduction in mortality.

Future work will be mainly focused on a further increase in graph complexity, including population information that was unavailable for this study, such as gender, religious beliefs, different vaccine efficiency and effectiveness, and others.

We also plan to apply the developed model to other cities in RS, as well as to the whole population of RS, and in the final stage to other countries, if their population data are available.

## Supplementary Materials

The following are available online at https://www.mdpi.com/article/10.3390/ijerph18126217/s1, Figure S1: Simplified workflow of SEIR-V (Susceptible, Infectious, Recovered—Vaccinated) graph model. Figure S2: The cumulative number of the infected and total reduction in mortality during general population vaccination. Scenario 1a) minimized number of diseased, 2a) minimized number of infected. Figure S3: The cumulative number of the infected and total reduction in mortality during general population vaccination. Vaccination scenarios based on the number of individuals’ contacts: 1b) minimized number of diseased, 2b) minimized number of infected.
