# Supplementary material for: Modelling Voluntary General Population Vaccination Strategies during COVID-19 Outbreak: Influence of Disease Prevalence
Source: Int J Environ Res Public Health. 2021 Jun 8;18(12):6217. doi: 10.3390/ijerph18126217 (PMC8229990; doi:10.3390/ijerph18126217)
Supplement: Supplementary file 1 [file ijerph-18-06217-s001.zip › ijerph-1207911-supplementary/Supplementary material File S1.pdf]

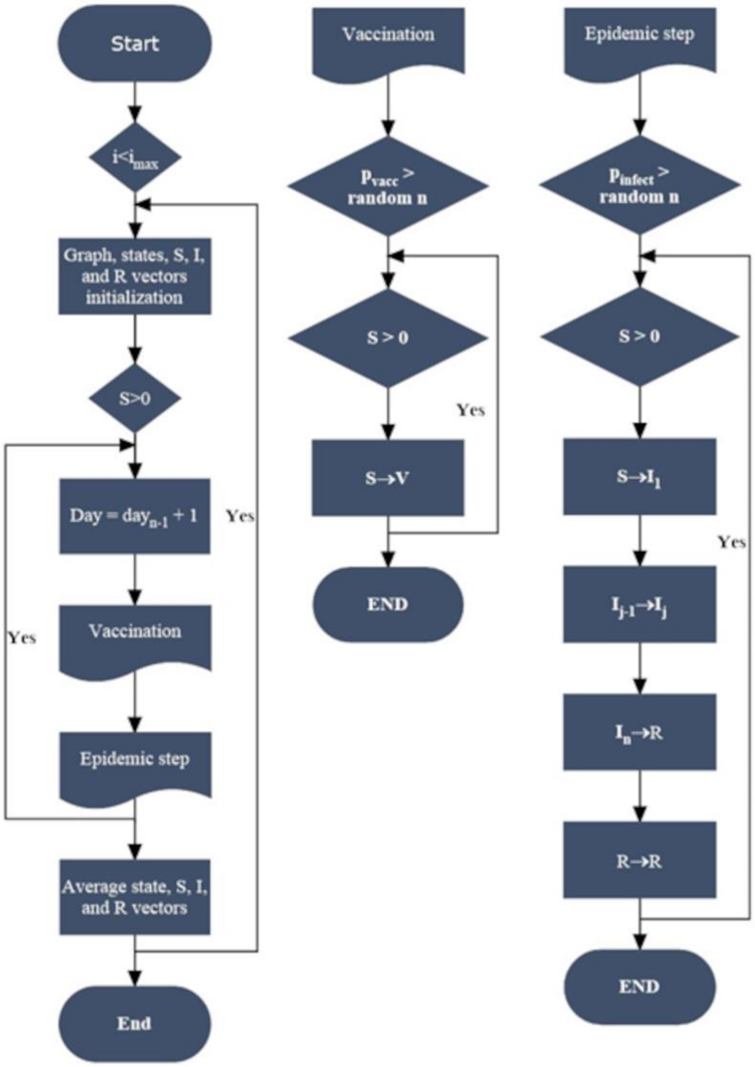

Figure S1: Simplified workflow of SEIR-V (Susceptible, Infectious, Recovered – Vaccinated) graph model

SEIR-V model workflow: Monte Carlo loop is started at the beginning of the simulation and performed until i_max number of Monte Carlo steps is reached. Arrays containing initial numbers of suspectable/exposed, S, infected, I, and recovered, R, individuals along with current stage of infection for each individual are initialized. If the number of suspectable/exposed S is larger than 0 simulation is advanced for 1-day and vaccination and epidemic steps are performed (these two steps are further explained in panels 2 and 3). The loop is advanced until there is no more suspectable cases. On exit from the Monte Carlo loop averaged arrays S, I, and R and averaged epidemic stage for each infected individual are calculated.
