# Supplementary material for: Modelling Voluntary General Population Vaccination Strategies during COVID-19 Outbreak: Influence of Disease Prevalence
Source: Int J Environ Res Public Health. 2021 Jun 8;18(12):6217. doi: 10.3390/ijerph18126217 (PMC8229990; doi:10.3390/ijerph18126217)
Supplement: Supplementary file 1 [file ijerph-18-06217-s001.zip › ijerph-1207911-supplementary/Supplementary material File S2.pdf]

It can be seen that the first scenario leads to a faster increase and a higher final cumulative number of infected. In other words, prioritizing vaccination of social groups with large numbers of contacts delays epidemic peak and lowers the total number of COVID-19 infected, Figure S2. The opposite trend is observed for the number of COVID-19 deaths. Namely, prioritizing vaccination of most vulnerable groups (scenario 1a)) reduces mortality by 57% compared with 50% in the case of prioritizing groups with the most contacts. It is important to underline that reduction of mortality is calculated as a relative difference between mortalities in scenarios with vaccination and without vaccination.

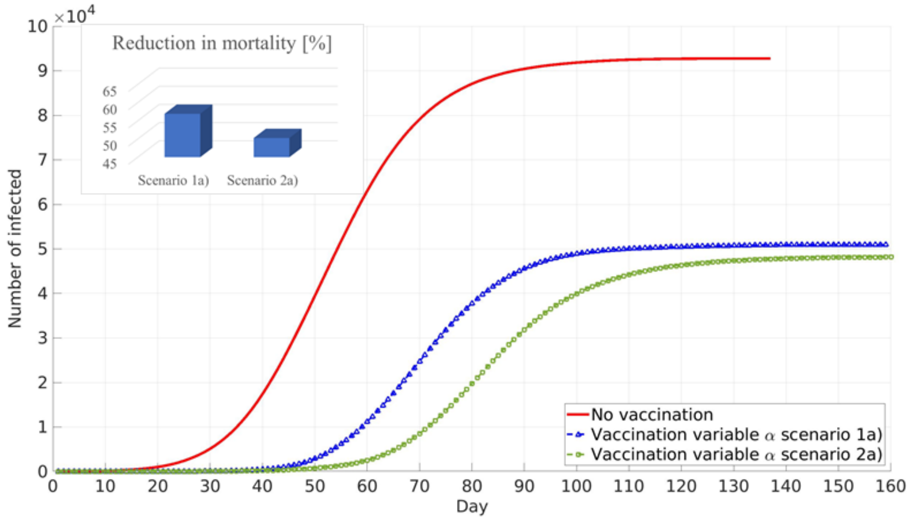

Figure S2. The cumulative number of the infected and total reduction in mortality during general population vaccination. Scenario 1a) minimized number of diseased, 2a) minimized number of infected.

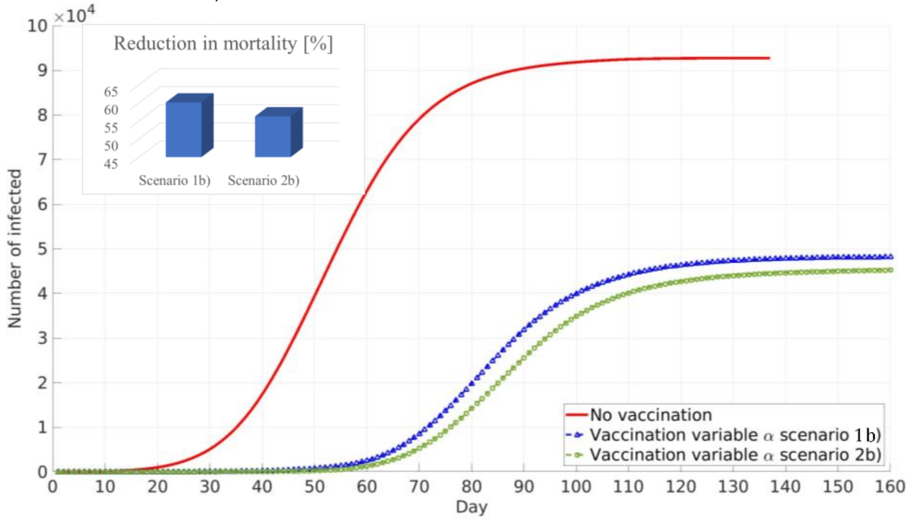

Figure S3. The cumulative number of the infected and total reduction in mortality during general population vaccination. Vaccination scenarios based on the number of individuals' contacts: 1b) minimized number of diseased, 2b) minimized number of infected.

It can be seen, in Figure S3, that adopting a vaccination strategy based on numbers of social contacts reduces the final number of infected by about 4% for both prioritization approaches compared with previous scenarios 1a) and 2a). More importantly, the sharp increase in the number of infected corresponding to the epidemic peak is delayed for 14 days when applying a strategy for minimizing numbers of deceased and for 10 days when applying a strategy for minimizing the number of infected, Figures S2 and S3. The decrease in mortality is higher than in previous scenarios, for 7% when switching from scenario 1a) to scenario 1b) and for 6% when switching from scenario 2a) to scenario 2b).
